# Exploration of physical activity knowledge, preferences and support needs among pulmonary hypertension patients

**DOI:** 10.1371/journal.pone.0277696

**Published:** 2023-01-18

**Authors:** Ciara McCormack, Brona Kehoe, Sarah Cullivan, Noel McCaffrey, Sean Gaine, Brian McCullagh, Niall M. Moyna, Sarah J. Hardcastle

**Affiliations:** 1 School of Health & Human Performance, Dublin City University, Dublin, Ireland; 2 Department of Sport & Exercise Science, Waterford Institute of Technology, Waterford City, Ireland; 3 National Pulmonary Hypertension Unit, Mater Misericordiae University Hospital, Dublin, Ireland; 4 ExWell Medical, Dublin, Ireland; 5 Department of Sport and Physical Activity, Sheffield Hallam University, Sheffield, United Kingdom; 6 Institute for Health Research, University of Notre Dame, Fremantle, Western Australia, Australia; Medizinische Universitat Graz, AUSTRIA

## Abstract

**Objective:**

Physical activity (PA) is an established adjunct therapy for pulmonary hypertension (PH) patients to mitigate PH symptoms and improve quality of life. However, PA engagement within this population remains low. This study investigated PH patients’ knowledge of PA, recalled advice, exercise preferences and PA support needs.

**Methods:**

Semi-structured interviews were conducted with 19 adults (mean age 50 years; SD ±12 years) diagnosed with PH, living in Ireland. Interview scripts were digitally recorded and transcribed verbatim. Thematic analysis was used to analyse the data.

**Results:**

Four key themes were identified: Lack of PA knowledge; exercise setting preference; accountability and monitoring; and clinician delivered PA information and guidance.

**Conclusion:**

This study found that PH clinicians provide suboptimal PA advice, yet patients desired clinician-delivered PA guidance. Home-based exercise was preferred with monitoring and external accountability deemed as important to facilitate sustained engagement.

**Practice implications:**

PH clinicians are well positioned to play a critical role in assisting and empowering PH patients to engage in PA. Providing training and education to PH clinicians regarding exercise prescription may be beneficial. Further research is needed to evaluate the feasibility and efficacy of home-based exercise interventions to improve quality of life and physical activity in PH.

## Introduction

Pulmonary hypertension (PH) is an incurable disease of the pulmonary circulation, characterized by progressive pulmonary vascular remodelling and increased right ventricular afterload, resulting in impaired cardiac function. Progressive dyspnoea and exercise limitation are the clinical features of PH. These debilitating symptoms have negative consequences on patient quality of life (QoL), independence and physical activity (PA) levels [1–3]. Due to the expansion of treatment options in the preceding two decades, PH has transformed from a disease with a considerably poor prognosis to one where patients live for many years [4,5]. In light of this increased longevity, there has been a greater emphasis on a holistic approach to PH management [6], particularly in relation to optimising QoL and improving daily functioning.

Since 2019, PH guidelines have recommended PA and supervised exercise as treatment goals [7,8]. Despite these recommendations, research consistently demonstrates low PA participation and reduced QoL in this population [3,9–11]. Lower levels of PA has been associated with poorer aerobic fitness, [7] hemodynamic impairment [9] and reduced survival [12] among PH patients.

Given the benefits of PA for PH such as improves exercise capacity, QoL, muscle function and pulmonary circulation, patients would benefit from interventions aimed at promoting exercise engagement. However, a greater understanding of the psychosocial and behavioural factors that influence PA in PH is required to inform such interventions. Few studies have explored PA knowledge or the enablers, barriers, exercise preferences or support needs of PH patients necessary to design patient-centred interventions [13–15]. Previous PH research has identified the following barriers to exercise: low energy, interest and self-discipline [14]; uncertainties regarding the importance and safety of exercise [13] and dyspnoea, fatigue, and anxiety [13]. Fear of exercise (due to perceived overexertion, damage to health and breathlessness) also influences attitudes toward and engagement in PA amongst PH patients [15].

There is a dearth of research regarding patient knowledge, exercise preferences, and support needs for individuals with PH, that could facilitate the design of appropriate and acceptable exercise programmes [8]. In the only study to date, Chia et al, [13] found that education about exercise, qualified exercise personnel and access to PH specific exercise programmes facilitated exercise engagement in PH patients. To our knowledge no study has qualitatively explored PA knowledge, recalled advice or the exercise preferences and support needs of PH patients. Qualitative approaches are important to gain a deeper understanding of the influences on PA behaviour change [13].

Several theories have been developed to understand the process of PA behaviour change. The health belief model (HBM) is comprised of perceived susceptibility to disease, perceived benefits (of the treatment), perceived barriers, perceived severity and cues to action [16]. Accordingly, if an individual considers themselves vulnerable to a serious health threat and believes that PA will be effective in reducing the threat and that the benefits of PA outweigh the costs involved, they will engage in PA. The theory of planned behaviour focuses on the role of attitudes, social norms and perceived control and intention to explain PA behaviour [17]. Bandura’s social cognitive theory (SCT) [18] focuses primarily on self-efficacy (i.e., confidence to engage in PA) and outcome expectancies (i.e., confidence that PA participation will result in attaining desired outcomes), and the self-determination theory [19] focuses on the quality of motivation and the importance of satisfying three core psychological needs: that of autonomy, competence and relatedness. To date, there is no optimal model to explain PA behaviour change, and qualitative research is useful, by not taking a deductive approach (i.e., adopting a single theory) and approaching the topic with a broad perspective to help unravel which dimensions appear to be most important [20]. A qualitative approach that is not wedded to a particular theory is also helpful when there is a dearth of research on the topic, as is the case in exploring influences on PA in PH patients. Further, the inductive approach employed in our analysis demands that the emergent themes are data, rather than theory, driven [21,22]. The present study aimed to investigate PH patients’ knowledge of PA, recalled exercise advice, exercise preferences, and PA support needs.

## Methods

The current study conformed to the suggested recommendations of the standard for Reporting Qualitative research (SRQR) checklist [23] (S1 Appendix). The study adopted a social constructionist thematic analysis underpinned by a realist ontology [24].

### Participant recruitment

A purposive sampling strategy was used to recruit participants for this study. The inclusion criteria included adults with a formal diagnosed of PH (WHO Groups 1 and 4), as per ESC/ERS 2015 guidelines [25], on conventional PH therapy currently attending the National Pulmonary Hypertension Unit in Dublin, Ireland and with fluent English. Participants were recruited via the Pulmonary Hypertension Association of Ireland mailing list of subscribed PH patients (n = 105). A formal email invitation was sent to all subscribers, which included a patient information leaflet. One subsequent follow up email was administrated. Following an expression of interest, written informed consent was obtained and mailed to the research team. Ethical approval for this study was granted by the Dublin City University Research Ethics Committee (DCUREC/2020/155).

### Data collection

Semi-structured interviews were used to obtain data. Interviews were conducted via a virtual platform, with a duration between 35–55 minutes (between July and August 2020). An interview guide was developed based on previously published work [20,26], and consisted of open-ended questions related to the study aims. Examples of some of the questions include; “*What would assist you to be more physically active*”; “*Have you received any advice or support regarding PA*?”; “*In an ideal world*, *what would the perfect PA program look like for you*?”. Interviews were co-lead by CMCC and SH, digitally recorded with permission and transcribed verbatim. The aim was to achieve a sample size of 15–20 in line with similar qualitative studies [20,26,27].

### Data analysis

Data was analysed using a six-step thematic analysis approach [28]. The first step involved reading all of the scripts numerous times to become immersed in the data. The second step involved initial systematic coding. The third step involved the collation of coded data into potential themes. Inductive analysis was utilized during this process to identify themes that emerged from the data. A ‘thematic map’ was developed in the fourth step. This was a two-phase process. Highlighted themes were reviewed and checked for coherency, and subsequently reviewed within the content of the data set to ensure that they accurately reflected the opinions and experiences of study participants. The fifth step, focused on refining and defining of themes. The final step involved the selection of appropriate extracts (to exemplify themes), discussion of the analysis (to ensure themes are distinct) and final review of all coded data.

In order to establish credibility in data collection and analysis, several steps were employed. Firstly, two researchers (with insider and outsider perspectives) conducted the interviews and independently analyzed the data. The primary researcher (CM) has experience in qualitative research approaches with a particular focus on PH which allowed for a mutual understanding of disease, limitations and terminology used by participants (insider perspective). The second researcher (SH) has extensive experience in qualitative research alongside a wealth of knowledge in health psychology and behaviour change but no prior association with PH patients (outsider perspective). In the process of data analysis there were no predetermined themes prior to data collection and inductive inference was used in the identification of themes and data interpretation.

Data collection ceased at the point when no new information was provided, and data saturation was obtained [29]. To determine this the two interviewers discussed interviews in real time, following each interview, with notes taken concerning themes arising from each interview. In this way, researchers were able to make judgements on common themes arising and whether any significant new information was arising from further interviews. Data saturation was achieved after the 15^th^ interview, however, to verify this decision and to encapsulate a more diverse sample, a decision was made to conduct the remaining four interviews since participants had already consented to take part and research in this field is limited. To further demonstrate credibility a rigorous approach to data analysis was ensured by the following steps:(i) a detailed description of the steps taken to conduct the thematic analysis, (ii) independent and comprehensive coding by two researchers thereby broadening the possible interpretations and (iii) including “thick description” via the use of extensive quotations to allow readers to evaluate the credibility of the interpretation” through the use of extensive and direct quotes to allow the reader the opportunity to evaluate the interpretations (i.e., the link between data extracts and theme labels) [22].

## Results

A total of twenty-six expressions of interest in participation were received. Two individuals did not meet the inclusion criteria, two were unavailable on specific interview dates and three did not respond to the confirmation email. Nineteen adults with PH participated in the study. Thirteen (68%) were female, the mean age was 50 (±12) years, 79% (n = 15) had a diagnosis of pulmonary arterial hypertension (PAH) and 21% (n = 4) a diagnosis of chronic thromboembolic pulmonary hypertension (CTEPH). Participants’ characteristics are described in Tables 1 and 2.

**Table 1:** Baseline characteristics.

Baseline Characteristics: n	19
Age (Years): mean ± SD	50 ± 12
Gender; Male: n (%)	6 (32)
PH WHO Group n (%)	
Group 1 PAH	15 (79)
Group 4 CTEPH	4 (21)
Duration of diagnosis (years): mean ± SD	8 ± 4
EmPHasis-10 score: mean ± SD	32± 11
Highest Level of Education: n (%)	
Primary School	1 (5)
Secondary School	4 (21)
Diploma/Certificate	10 (53)
Undergraduate	3 (16)
Postgraduate	1 (5)
Current Employment Status: n (%)	
Unemployed due to medical illness	13 (68)
Retired	3 (16)
Full time employment	3 (16)
Attended a community-based Exercise Programme n (%)	2 (10.5)

Demonstrates the baseline characteristics of patients included in this study. Abbreviations: SD = standard deviation; PH = Pulmonary Hypertension; WHO = World health organisation; PAH = pulmonary arterial hypertension; CTEPH = chronic thromboembolic pulmonary hypertension.

**Table 2:** Individual participant characteristics.

Pseudonym	Age	PH Group	Years Since diagnosis	Emphasis-10 Score (Total)
John	36	CTEPH	8	34
Paul	66	CTEPH	5	8
Mary	61	PAH	16	17
Shane	44	PAH	10	34
Emer	39	CTEPH	7	23
Amy	51	PAH	6	42
Hannah	39	PAH	5	43
Michael	47	CTEPH	7	14
Ann	67	PAH	15	26
Dorothy	54	PAH	3	41
Peter	52	PAH	6	33
Nicola	38	PAH	7	35
Rebecca	43	PAH	4	36
Rachel	48	PAH	15	36
Susanne	44	PAH	10	24
Paddy	76	PAH	4	44
Breda	63	PAH	7	37
Laura	40	PAH	4	36
Catherina	57	PAH	11	44

Participant pseudonyms and individuals characteristics. Abbreviations: PH = Pulmonary Hypertension; PAH = pulmonary arterial hypertension; CTEPH = chronic thromboembolic pulmonary hypertension, 6MWD: 6 minute walk distance. Epmhasis-10 is a PH specific measure of quality of life.

Table 3 displays participant characteristics compared to the Irish PH population with the latter data taken from a recent publication on incidence and outcomes of PH in Ireland by Cullivan et al. [30]. The comparison indicates that our Group 1 the study population is similar to the general Irish PH population in terms of age, gender distribution and functional class. In relation to Group 4, the present study may have recruited a younger cohort, however the fewer proportion of females is this group is consistent with the wider PH population.

**Table 3:** Study participants characteristics compared to the Irish PH population.

	Study PH Group 1 Population	Irish PH Group 1 Population	Study PH Group 4 Population	Irish PH Group 4 Population
**Subjects (n)**	15	163	4	67
**Gender; female (%)**	12 (80%)	125 (77%)	1(25%)	29(43%)
**Age (years)**	51 ± 11	56 ± 15	43±13	61 ± 16
**WHO Function class: % I/II/III/IV**	1/4/7/3	1/29/56/14	1/2/1/0	1/28/65/7

Study participants characteristics compared to Irish PH population. Abbreviations: PH = Pulmonary Hypertension, WHO; World health organisation.

Data analysis identified four key themes: Lack of PA knowledge; exercise setting preference; accountability and monitoring; and clinician delivered PA information and guidance. Participant quotes are followed by a pseudonym and the individual’s age. In line with Sandelowski, pronouns will account for indeterminate quantities where “most” implies approximately 75%, “several;” implies approximately 50%, and a “few” implies approximately 20% of the sample [31].

### Lack of PA knowledge

A lack of knowledge concerning the importance of, or benefits of PA was evident among the participants. This appeared to stem from a lack of information and advice provided to them at their PH clinic; “*Not any specific advice really*, *it’s more to do with my treatment*, *my symptoms*. *They haven’t really gone into the whole area of exercise really*” (Michael, 47). Despite regular 6MWTs in scheduled clinic visits, participants recalled minimal discussion regarding aerobic capacity or ways to improve it: “*I find they do it and they tell you what you got and how many meters and they might compare it to the last time*, *but I have never found that they have gone into detail on how to change things around at all” (Susanne*, *44)*.

Information and guidance regarding exercise from the PH clinical team was lacking in most instances; “*There’s no guidelines there at all*” (John, 36). Some had searched the internet for exercise information but recognised that such information may not be trustworthy: “*Anything I find out is stuff I find out myself if I go online… Again*, *you don’t know how much of that to trust… because it keeps changing and it is only Dr*. *Google*. *It’s not your own doctor*. *I would prefer it came from someone that I knew I could trust*, *and they knew my condition*” (Amy, 51).

Suboptimal awareness and knowledge of published PA guidelines was evident among the participants. With the exception of one participant, most were unaware of PA guidelines: “*No apart from getting half an hour of exercise every day that’s all I know*” (Rachel, 48) and another mentioning daily steps: “*No*, *you hear 10*,*000 steps a day and that’s about all*. *I don’t really know what we’re meant to be doing or what a healthy person is meant to be doing’* (Susanne, 44).

The lack of PA knowledge was not only related to exercise dose but also to concerns of safety. The importance of exercise programmes that are “*specific to people with PH*” (Ann, 67) and safe were consistently emphasised by participants. Reassurances regarding the safety of exercise was important to almost all participants; *“I would like to enjoy my exercise and do it but knowing that you were in safe parameters*” (Ann, 67), with many alluding to the fact that it would encourage them to engage in exercise; “*if I know it’s safe it would motivate me to do it because I know I’m not putting myself at risk*.” (Rachel, 48). An individualised approached may help mitigate the fear associated with exercise for some participants: “*I think the fear factor*… *is somewhat mitigated if you have some kind of a guideline or parameter that gives you an idea that this would probably be o*.*k*. *for your stats*, *your weight*, *your height*, *your PH and all that stuff*, *I think that would be really helpful*.*”* (Emer, 39).

### Exercise setting preferences

Many participants in this study reported a preference for home-based exercise rather than alternative settings such as gym or centre-based; “*I prefer to do it at home*” (Hannah, 39) and “*I would certainly like to start something at home*” (Mary, 61). This finding did not appear connected to COVID-19 lockdowns or concerns of contracting the virus. Two participants reported that they had previously attended a community-based exercise programme and considered it inappropriate for PH patients and travel time was a burden for them; “*But what I found was that it wasn’t particular to pulmonary hypertension*. *It was for people with COPD…I put everything into it but as the course went on*, *I declined during the exercises*. *I would be fainting and that kind of thing because it wasn’t specific to pulmonary hypertension… The travel to and from took a lot out of me too”* (Ann, 67). A home-based setting appealed to many as it provided them with maximum flexibility; “*it’s not putting pressure on myself*, *I can do it on my own time*, *so if I feel I have the energy to do it I can*” (Hannah,39). Home-based PA was also considered useful in overcoming barriers such as the weather and energy: “*I would definitely prefer to be at home*. *If it’s raining outside or I’m too tired to get dressed I can still do it at home*” (Amy, 51).

For several participants, travelling to a centre or a gym to complete exercise was deemed to be energy consuming and a burden;*”Just going into a gym would make it instantly less likely to happen… Getting up*, *dressed*, *getting into the car*, *by the time you get somewhere you are almost already exhausted anyway*. *Don’t tell me to go to a gym because I know I won’t*” (Rebecca, 43). For others the task of travelling was a potential barrier for them; “*I’d be exhausted having to get 2 or 3 buses there and then the same back*” (Nicola, 38), especially if the activity could be done at home; “*If I have to go travel halfway across the city to some place to do a half an hour of god knows what*, *aerobics*, *I mean I could just do that at home*” (Shane, 44).

Interestingly many participants also preferred exercise in their home environment because it was comfortable and viewed as a “safe” place; “*I have the comfort at home*… *home is safe…”* (Emer,39); “*That means I have to leave the house and be out and if I have a bad reaction*, *I’m out*. *Whereas if I am exercising at home and I stop then I am still at home*” (Rebecca,43). Additionally, those who preferred home-based exercise highlighted the benefits of family support; “*My family are great*…*They would be doing it with me*, *they would be so supportiv*e” (Rebecca, 43) and “*if my husband was here…I’d be more inclined to want to get out for a walk*” (Nicola, 38).

Conversely, a few participants reported a preference for facility-based setting. This setting was appealing primarily for motivation purposes rather than safety concerns; “*motivation would be a problem when I’m left on my own…so maybe if you were going somewhere*, *it would keep you motivated*” (Susanne, 44) and “*being supervised by a proper person*, *a physio or an instructor*, *that they can give you great encouragement and its very rewarding when you do it*” (Paddy,76). Only one participant referred to perceived greater safety exercising in a hospital setting: “*If I was in the hospital and something happened*, *well then I’d be in the right place for medical attention*…*I would feel much more secure and much safer*” (Peter,52).

One participant suggested a blended approach, with an exercise program that was initiated in a supervised setting and then transitioned to the home environment; “*having it supervised would be good that you have somebody overseeing what you are doing*, *but maybe just at the start… to get you to know what you could do safely… and then maybe have stuff you could do at home”* (Susanne, 44).

Focusing on exercise activities, study participant expressed a desire to engage in primarily aerobic exercise; “*I think walking*” (Breda, 63); “*I love swimming*” (Dorothy, 54); “*An exercise bike would interest me*” (Rachel, 48). Several participants highlighted a reduction in muscle mass since PH diagnosis and wished to address this with strengthening exercises; “*I know I have muscle wastage now*” (Mary, 61); “*it would be nice to have guidelines*, *not lifting weights as such but something to strengthening up*” (Susanne,44). Some reported previously avoiding strengthening activities due to safety concerns; “*I don’t do any weights or anything like that*. *I suppose I shied away from that thinking I might harm myself or damage myself in some way so I don’t lift any weight” (Michael*, *47)*.

### Accountability and monitoring

Accountability and external monitoring was considered important for many participants, to foster exercise engagement. Many participants spoke about the desire to be accountable to someone; “*I think that’s probably my problem is having the accountability*… *If I feel that there’s somebody checking up on me*, *maybe I feel I’ll do it better… sometimes we need that little push*.” (Susanne, 44);”*If I know someone is watching me*, *if I know I have to answer to someone*, *I will try more*” (Amy, 51). Participants felt that a regular review was important but not to feel overwhelmed by it; “*You wouldn’t want someone there 24/7 obviously but to check in once in a while would be good”(Rebecca*, *43)*.

Participants expressed how external monitoring by health professionals and self-monitoring tools would be helpful to encourage PA engagement, improve motivation and assess progress. Some participants referred to current use of such devices, to quantify activity levels and check vital signs, and suggested that such tools may be valuable in future exercise programmes; “*I have this app on my phone to monitor my activity and so I try and get up to 2 and a half or 3 kilometres of walking a day*”(Micheal, 47) and “*I monitor my heart rate and my oxygen levels*” (Rachel, 48) and “*I think some kind of comfort*, *like the way the Fitbit works is good*, *because I think it would make you feel more comfortable slowly pushing yourself because you would feel in a safe environment and you can check that something is monitoring you*” (Emer, 39).

Some participants conveyed that clinic visits would be a suitable time to incorporate exercise assessments and would incentivise them to persist with the activities; **“***I would not like to go in [to clinic] and say I haven’t done any of the work*. *That would encourage me as well*. *I would hate anyone to go to the trouble of showing me what to do and not do it” (Rachel*, *48)*.

Almost all participants desired individualised information regarding the type, frequency and intensity of specific activities; “*If I have guidance from you guys on what a good weights programme or even an aerobic programme*, *how much*, *how often*, *how hard all that*, *I would love that*. *More guidance from professionals*. *That would be helpful for somebody like me*. *Just a bit of guidance to give me confidence in what I’m doing is good*, *not damaging*” (Paul, 66).

### Clinician delivered PA information and guidance

The authority of, and trust in their PH clinicians was a key theme concerning the delivery of PA information and advice. Most participants desired exercise advice directly from their clinician; “*I prefer it came from someone that I knew I could trust*, *and they knew my condition*…*If the clinicians said something 100%*, *I would follow it*.” (Amy, 51). A high level of trust was expressed by most participants; “*You have to put all of your trust into them for everything so this {exercise} would be another area… I trust my consultant 110%*, *if he said to me do this I would do it*” (Emer, 39) and “*I think anything Professor would say to me I would listen to 100% because I have huge respect for him… I would give anything a go that they would say*” (Ann, 67). Participants trusted their PH clinician and expressed confidence in their advice, which encompassed recommendations regarding PA and exercise.; “*So*, *I’d say if they [PH clinician] are giving advice to you they would know… you’d feel more confident maybe if they are saying you can do whatever*” (Breda, 63); *“If the professor told me that I was able to it*. *If he said to me you could do A*,*B*,*C*,*or D I would go by that because he knows me*, *he knows how I handle it*. *If he told me that I could do this*, *I would be very confident that that would be the case”* (Rachel, 48). Interestingly, even a participant that was resistant to exercise indicated that he would participate if recommended to do so by his PH clinicians: “*Trust me*, *if there was an exercise programme there and was recommended by the professional such as the professor*, *I would definitely try it without a doubt*” (Peter, 52).

PA information or advice was considered valuable if provided by any member of the PH clinical team or a professional that they were referred to by their specialist team; “*Something that is approved by the team rather than necessarily delivered by them*” (Paul, 66) and *‘It would be great if there was a leaflet or just a couple pages on it and then even if you went back then after six months*, *they’d give you bit more or point you in the direction of someone*” (John, 36). One participant described that information is more impactful when initially explained verbally by the PH clinicians; “*It’s better to be spoken then written*, *I would say*, *in the beginning*. *People get a leaflet*, *they just leave the leaflet*. *If they [PH Clinicans] are speaking about it*, *they will remember something about that*” (Laura, 40).

A multidisciplinary team (MDT) approach was suggested by many, with “*exercise experts*”, “*other PH professionals*” and/or “*physiotherapist*” as additional member of the clinical team, who could provide advice and/or deliver a programme; “*I think we would need nearly a physiotherapist to be part of that really to take over the exercise part of it rather than the nurses because they’re so busy*” (Ann, 67).

## Discussion

This is the first study to explore PA knowledge, exercise preferences and support needs amongst PH patients. The current study identified suboptimal PA education and advice from their PH team and a desire for clinician-delivered PA information and exercise recommendation. Participants had a preference for home-based exercise, with strategies such as monitoring and external accountability to ensure engagement and sustain motivation.

In the present study participants did not recall receiving PA advice by their PH clinician team. This finding is contrary to that of Chia et al., [13] who found that 77% of PH patients reported receiving general PA information and more than half (56%) receiving specific PH exercise guidelines. Whilst recollections may differ, it is perhaps unsurprising that PH patients may not receive PA advice since the use of exercise as an adjuvant treatment for PH is relatively new. Additional barriers to exercise promotion include a lack of PH specific exercise guidelines [8] and uncertainty regarding the optimal exercise prescription for PH [32].

The authority of, and trust in, the PH clinicians represents an important and novel finding. Many participants indicated that they would engage in an exercise programme if advised to by their PH clinician. Given their position of authority, PH clinicians are ideally placed to provide exercise information to patients during outpatient consultations and facilitate a conversation concerning PA. Research in other clinical cohorts has demonstrated promise for the effectiveness of brief advice by healthcare professionals on patient PA engagement [33,34]. A collaborative approach in the delivery of exercise programmes using a MDT and exercise specialists was also considered acceptable and desirable amongst participants.

A key finding in this study was the preference for home-based PA rather than a gym or facility-based programmes. Participants highlighted the benefits of a home-based setting, which offered flexibility, omitted travel, removed weather-related barriers and provided a safe and comfortable exercise environment. Only a few participants preferred a supervised setting and this was driven by a desire for motivation and external support rather than safety concerns. These findings contrast prior research where most participants reported a preference for a supervised and structured exercise programme this may be due to difference geographic location of the population previously the majority of participants resided in Australia or New Zealand (61%) [13]. The present study suggests that home-based PH exercise programmes may be acceptable to PH patients. To date, very few PH exercise training studies have assessed the acceptability, safety and efficacy of home-based interventions, but display promising results [35,36]. It is worth noting that exercise preferences may vary from patient to patient, therefore it is important to tailor interventions according to individual preferences including setting and type of exercise.

Finally, the importance of accountability and monitoring was highlighted in this study. External factors that enhanced motivation were important for participants. Potential internal barriers included a lack of self-discipline, which is consistent with previous studies [13]. These finding suggest a potential role for clinicians to provide external accountability and monitoring of PA during outpatient appointments. Furthermore, interventions that target patient self-efficacy, motivation, exercise knowledge and self-regulation would be valuable. PH exercise training interventions typically do not employ behavioural change techniques [37] which should be addressed. The supplementary role of self-monitoring tools such as mobile applications and wearable devices was also highlighted as useful by participants in the present study.

The themes identified in the present study do not clearly align with a specific theory of behaviour change but lend support for the principle of triadic reciprocity in SCT. According to SCT, behaviour may be explained by reciprocal interactions between social-cognitive, behavioural and environmental influences [38]. For example, the theme of exercise setting preferences supports the reciprocal links of SCT in terms of the behaviour, and attitudes towards the behaviour (i.e., social-cognitive), being influenced by the environment (i.e., the exercise setting). The environment, in relation to the desire for support and guidance from clinicians, also appeared to influence knowledge of and attitudes towards PA including motivation (i.e., social-cognitive influences). Further research is warranted to help examine which theoretically framework(s) better explain PA behaviour in patients with PH. The results of the present study should be interpreted in an explorative/hypothesis-generating manner and further research is necessary to confirm or refute these exploratory findings.

### Practice implications

PH specialists have the authority, trust and opportunity to play a critical role in educating PH patients regarding the benefits of exercise, providing exercise prescriptions and addressing safety concerns. Notwithstanding time constraints, the opportunity to discuss PA could be integrated into the discussion of the 6-minute walk test (6MWT) results that is routinely performed at each clinic visit. PH teams may benefit from formal training and education to provide the specific, detailed information that they require to advise their patient cohort. This should include the concepts of exercise prescriptions and brief exercise counselling techniques, that could be incorporated into routine clinical practice. Furthermore, this study demonstrates a potential patient preference for home-based exercise programmes that will require further exploration regarding feasibility, efficacy and safety. Adequate infrastructure regarding personnel and telehealth technology is required to facilitate home-based exercise and requires MDT collaboration. Finally, the role of self-monitoring tools and wearable devices has been highlighted by this study cohort and alongside evidence-based behavioural change techniques should be considered for inclusion in future interventions to foster more autonomous forms of motivation and sustained behaviour change.

### Strengths and limitations

It is important to consider the limitations within this study. The absence of exclusion criteria must be noted as a limiting factor. Due to the voluntary nature of this study, those who agreed to participate may have a particular interest in PA and thus the results may not be reflective of the wider PH population. Furthermore, the results were derived from a predominantly female population, and therefore may not transfer to males. The national sample of participants is a strength of the study and captured the preferences from different geographical regions and outside of metropolitan areas in addition to a diverse age range (36-76years). Further research would be worthwhile to confirm or refute present findings which may not be representative of the wider PH cohort. It is also important to acknowledge the timeframe of the research in relation to the global pandemic, although not highlighted by participants within the study, it may potentially have influenced their viewpoints in terms of exercise preferences.

### Conclusion

The study highlights suboptimal knowledge of PA and inadequate advice provided to participants by their PH clinical team. Participants expressed a strong desire to receive such guidance and support from their treating clinicians. A preference for home-based training with monitoring was highlighted. This study underscores the critical role of PH clinicians regarding PA education and PA prescriptions during routine clinical assessments. Findings from this study suggest that future interventions should offer specific, individualised information on exercise, and support and monitoring in a home-based setting.

## Supporting information

S1 Appendix (DOCX)
